# YAP-mediated mechanotransduction in urinary bladder remodeling: Based on RNA-seq and CUT&Tag

**DOI:** 10.3389/fgene.2023.1106927

**Published:** 2023-01-20

**Authors:** Xingpeng Di, Liyuan Xiang, Zhongyu Jian

**Affiliations:** ^1^ Department of Urology, Institute of Urology (Laboratory of Reconstructive Urology), West China Hospital, Sichuan University, Chengdu, Sichuan, China; ^2^ Department of Clinical Research Management, West China Hospital, Sichuan University, Chengdu, Sichuan, China

**Keywords:** RNA-seq, CUT&Tag, YAP, bladder remodeling, mechanotransduction, fibrosis

## Abstract

Yes-associated protein (YAP) is an important transcriptional coactivator binding to transcriptional factors that engage in many downstream gene transcription. Partial bladder outlet obstruction (pBOO) causes a massive burden to patients and finally leads to bladder fibrosis. Several cell types engage in the pBOO pathological process, including urothelial cells, smooth muscle cells, and fibroblasts. To clarify the function of YAP in bladder fibrosis, we performed the RNA-seq and CUT&Tag of the bladder smooth muscle cell to analyze the YAP ablation of human bladder smooth muscle cells (hBdSMCs) and immunoprecipitation of YAP. 141 differentially expressed genes (DEGs) were identified through RNA-seq between YAP-knockdown and nature control. After matching with the results of CUT&Tag, 36 genes were regulated directly by YAP. Then we identified the hub genes in the DEGs, including CDCA5, CENPA, DTL, NCAPH, and NEIL3, that contribute to cell proliferation. Thus, our study provides a regulatory network of YAP in smooth muscle proliferation. The possible effects of YAP on hBdSMC might be a vital target for pBOO-associated bladder fibrosis.

## Introduction

Fibrosis are characterized by excessive proliferation and transformation of fibroblast and extracellular matrix (ECM) deposition. The annual incidence of fibrotic diseases is 4,968 per 100,000 person-years, resulting in a severe burden on patients (Zhao et al., 2020). Fibrotic diseases include multiple organs, such as the liver, kidney, heart, lung, and urinary bladder (Henderson et al., 2020). Moreover, the abnormal activation of myofibroblast triggered by transforming growth factor, platelet-derived growth factor, and fibroblast growth factor is identified as a major alteration in fibrosis (Zhao et al., 2022). Hence, a large number of patients suffer from fibrotic diseases that need more effective therapies indeed.

The fibrotic process is initiated by tissue injury. Moderate injury often leads to a tissue repair process. In contrast, severe or long-term wound-healing strategies can cause fibrotic changes to tissue and organ. The etiologies vary in different situations, including hypertension, myocardial infarction, acute or chronic infection, diabetes, alcohol damage, radiation, and others (Rockey et al., 2015). In the urological system, fibrotic processes are triggered in the kidney, ureter, and urinary bladder. Bladder fibrosis is often caused by partial bladder outlet obstruction (pBOO), cystitis, and radiation (Di et al., 2022). We have investigated that mechanical cues in pBOO, including hydrostatic pressure, fluid shear stress, stretching force, and ECM stiffness, activate the bladder fibrosis process.

Yes-associated protein (YAP) has long been recognized as an intracellular mechanical transducer (Dupont et al., 2011). As an important transcriptional co-activator, YAP is also a crucial downstream effector of the Hippo signaling pathway (Moya and Halder, 2019). YAP can respond to cell geometry, density, and substrate adhesion to promote the progression of fibrotic diseases. YAP has been commonly identified in cell renewal, cell differentiation, epithelial-to-mesenchymal transition, and fibrosis (Tang et al., 2013; Ohgushi et al., 2015; Lin et al., 2018). In addition, YAP has been investigated in many mechanical cues-induced organ remodeling, such as atherosclerosis (Wang et al., 2016), orthodontic tooth movement (Deng et al., 2021), nerve regeneration (Li et al., 2021), and others.

YAP can sense the change of stretch and ECM stiffness in the urinary bladder to promote downstream gene expression. Besides ECM deposition, bladder smooth muscle proliferation is an important pathological change in the compensatory stage of bladder fibrosis (Lai et al., 2019; Chen et al., 2020). Therefore, an RNA transcriptome sequencing (RNA-seq) of YAP-knockdown (YAP-KD) and YAP CUT&Tag of human bladder smooth muscle cell (hBdSMC) were performed to investigate the key genes and regulatory network of smooth muscle proliferation in bladder fibrosis. Chromatin immunoprecipitation sequencing (ChIP-seq) is used to study transcriptional factors and target DNA. The prediction through DNA segments indicates the interaction between transcriptional factors and downstream molecules. The CUT&Tag is an improved ChIP-seq with higher quality and can be conducted with only 10^5^ cells (Kaya-Okur et al., 2019). In fibrosis research, we aim to provide novel insights into YAP-associated smooth muscle proliferation.

## Materials and methods

### Cell line and cell culture

hBdSMCs cell line was purchased from ScienCell, United States (Cat No.4310), which was cultured with SMCM medium (ScienCell, United States, Cat No.1102) with fetal bovine serum (10%), streptomycin (100 μg/mL), penicillin (100 U/mL), and growth factor.

### YAP adeno-associated virus (AAV) infection

For YAP knocking down, YAP-AAV with short hairpin RNAs (shRNAs) was obtained from *GeneChem* (Shanghai). Cells were infected by AAV and vector at a multiplicity of infection of 100 for 8 hours with minimal toxicity.

### CUT&Tag sequencing

CUT&Tag sequencing was performed to analyze target genes of YAP. The sequencing technology was supported by Jiayin Biomedical Technology, Shanghai. The Raw Reads were sheltered by Trimmomatic software for Clean Reads (Bolger et al., 2014). Q20, Q30, and GC content parameters were used to assess the data quality (Supplementary Table S1).

### RNA sequencing

Transcriptome sequencing and analysis between the control and YAP-KD group was carried out with the assistance of *Bioprofile*, Shanghai. Total RNA was extracted with a standard protocol of RaPure Total RNA Kit (*Magen*, Guangzhou, Cat No. R4011-02). Q20, Q30, and GC content parameters were used to assess the data quality (Supplementary Table S1).

### Data analysis

The differentially expressed genes (DEGs) were clarified with the standard of adjusted *p* < 0.05, |log2FoldChange| ≥ 1. We intersected CUT&Tag sequencing with RNA-seq to obtain the common DEGs in both two sequencing for further analysis. To identify the function of DEGs, GO enrichment analysis and KEGG pathway enrichment analysis were performed with R package *clusterProfiler* (The R foundation; http://r-project.org; version, 4.2, United States). The protein-protein interactive (PPI) analysis of all DEGs was conducted with STRING (https://cn.string-db.org). The motif matching was performed with *HOMOR* software. *Cytoscape* software (version 3.9.1, JAVA version 11.0.6) was used to select hub genes.

## Results

### RNA-seq analysis of total DEGs for YAP-KD hBdSMC

**FIGURE 1:**
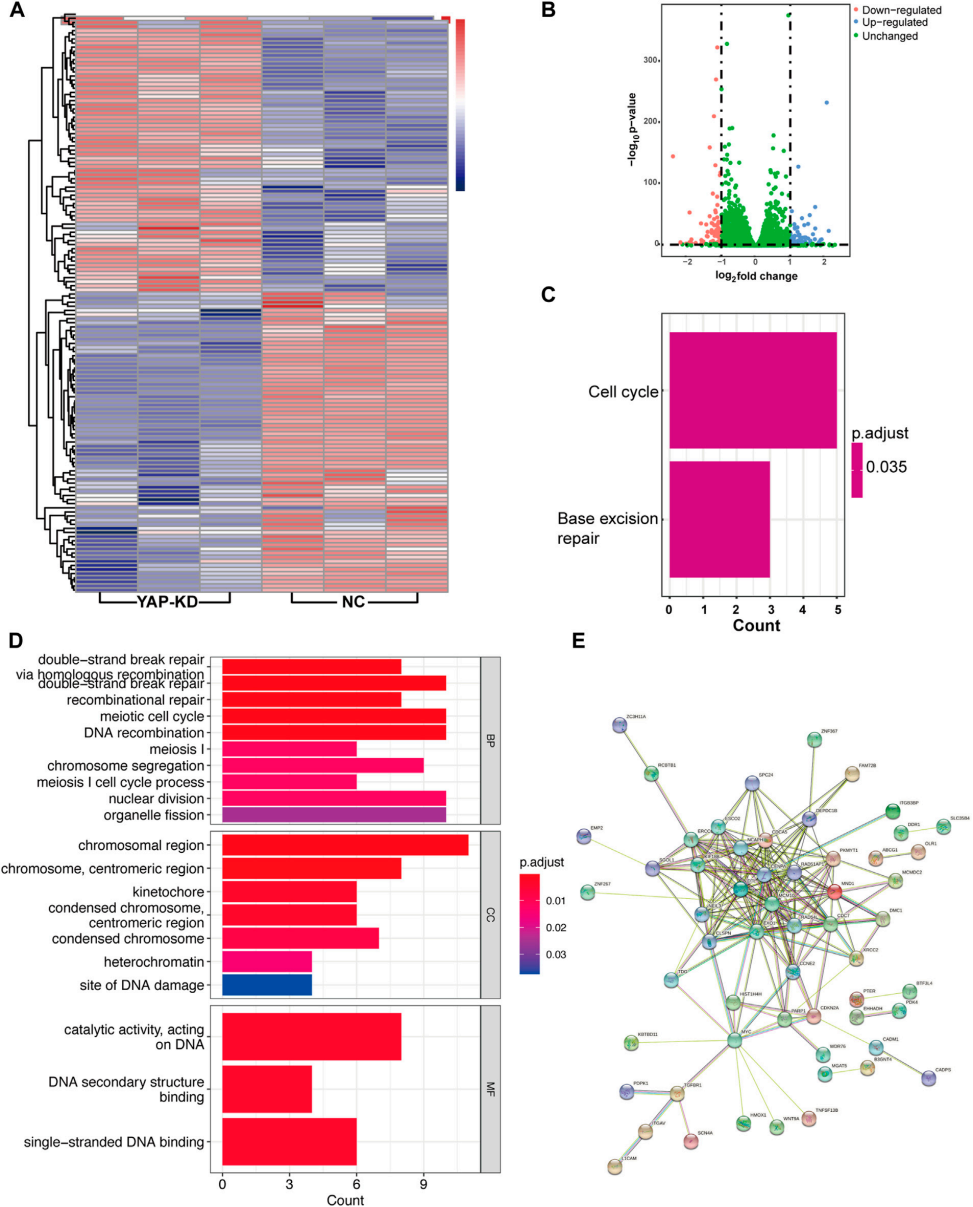
RNA-seq analysis of total DEGs for YAP-KD hBdSMC. **(A)**. The heatmap of YAP-KD and NC hBdSMCs. **(B)**. The volcano plot of DEGs. **(C)**. KEGG analysis of DEGs. **(D)**. GO analysis of DEGs. **(E)**. The PPI network of DEGs. hBdSMC: human bladder smooth muscle cell; KD: knock-down, NC: nature control, DEG: differentially expressed genes, PPI: protein-protein interaction.

To investigate whether YAP can regulate hBdSMC proliferation in bladder fibrosis, RNA-seq on the YAP-KD and nature control (NC) group with three duplicates. The results of RNA-seq indicated 141 DEGs in total, including 68 up-regulated DEGs and 73 down-regulated DEGs, which were presented in the heatmap and a volcano plot (Figures 1A, B). The top5 up-regulated and down-regulated DEGs were listed separately in Table1. To identify the function of DEGs, KEGG enrichment analysis was performed in Figure 1C showed that YAP regulated cell cycle and base excision repair pathways in hBdSMC. GO enrichment analysis was performed using all 141 DEGs. The results demonstrated that YAP engaged in DNA structure binding, meiotic cell cycle, recombination, and other proliferation-related cellular functions (Figure 1D). According to the DEGs acquired from RNA-seq, PPI analysis showed the interactions between proteins (Figure 1E).

**TABLE 1:** List of top 5 up-regulated and down-regulated DEGs of RNA-seq.

Gene_id	Gene_name	Expression_KD	Expression_NC	log2FC(KD/NC)	Adjusted P	Regulate
ENSG00000100292	HMOX1	314.9083048	75.52983456	2.059668088	2.35E-102	up
ENSG00000170801	HTRA3	16.46288202	6.991509256	1.234355134	8.50E-57	up
ENSG00000037749	MFAP3	1.594140556	0.475530677	1.724166201	4.07E-28	up
ENSG00000134717	BTF3L4	11.20840461	5.428414978	1.044609376	3.49E-25	up
ENSG00000173391	OLR1	1.924046244	0.64330886	1.565785182	2.42E-22	up
ENSG00000138448	ITGAV	13.09954398	28.48548158	−1.120115672	1.70E-141	down
ENSG00000143799	PARP1	13.70361237	30.55700841	−1.156366713	8.54E-119	down
ENSG00000122376	SHLD2	7.049022041	16.39803251	−1.216862024	1.18E-92	down
ENSG00000120253	NUP43	4.053020293	10.27518947	−1.339943896	1.79E-70	down
ENSG00000148841	NUP43	1.787120312	9.528147362	−2.408022075	3.95E-64	down

DEG, differentially expressed genes; KD, knock down; NC, nature control; FC, foldchange.

### The down-regulated DEGs regulatory network and gene functions

**FIGURE 2:**
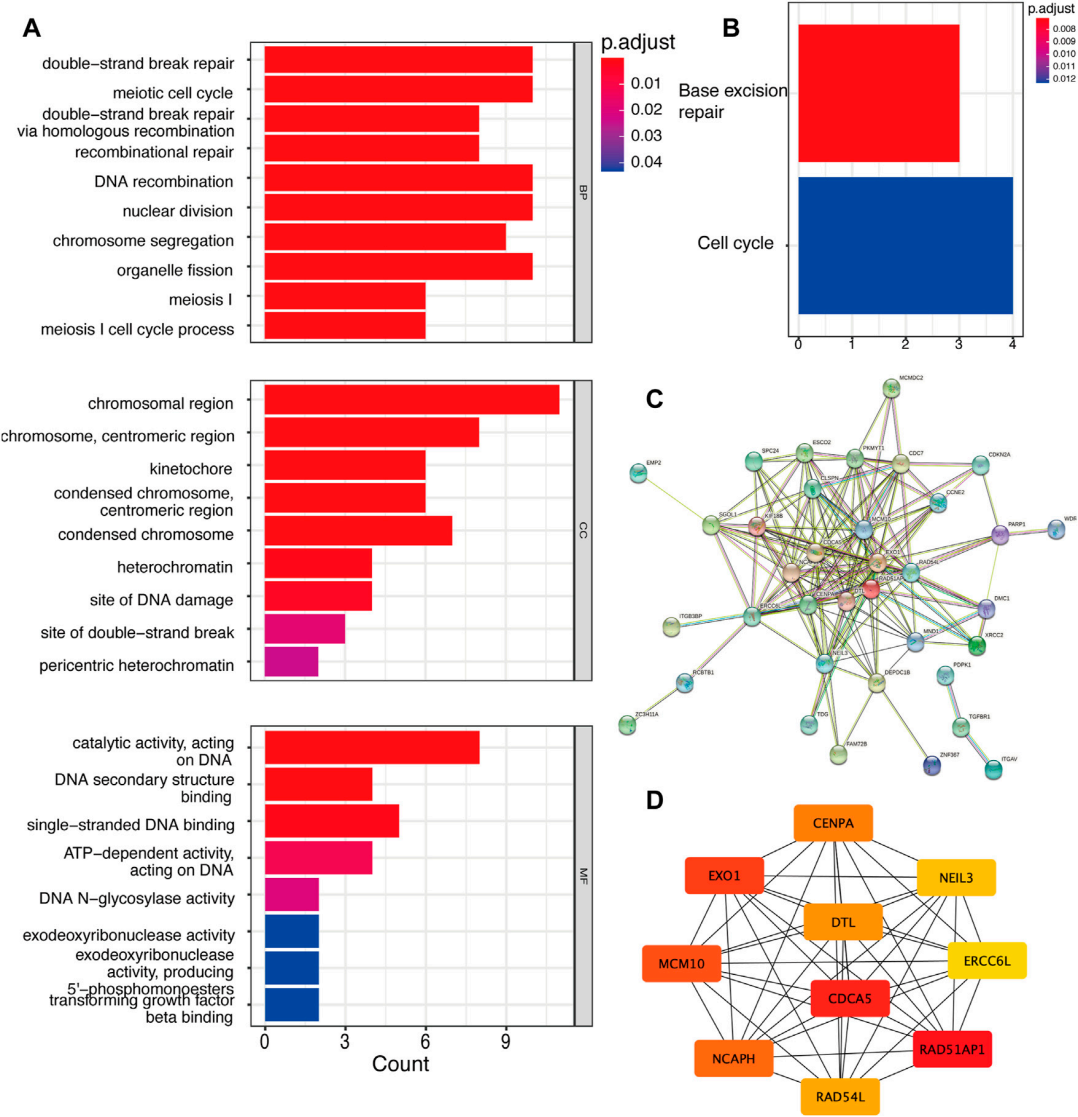
The down-regulated DEGs regulatory network and gene functions. **(A)**. GO analysis of down-regulated DEGs. **(B)**. KEGG analysis of DEGs. **(C)**. The PPI network of DEGs. **(D)**. Top 10 hub genes of down-regulated genes. DEG: differentially expressed genes, PPI: protein-protein interaction.

Apart from the analysis of total DEGs, we further analyzed up-regulated and down-regulated DEGs separately. Unfortunatey, we did not enrich enough function and signaling pathways in up-regulated genes *via* GO and KEGG analyses. Hence, we focused on down-regulated genes along with YAP-KD functions most in cell biology. GO results showed that YAP promoted the cell cycle, DNA binding, and TGFβ binding process (Figure 2A). KEGG results indicated that the DEGs enriched in base excision repair and cell process pathway (Figure 2B). The PPI network identified the interactions between down-regulated proteins (Figure 2C). The Maximum Climate Centrality (MCC) method was used to shelter the hub genes (Chin et al., 2014); the top five hub genes were RAD51AP1, CDCA5, EXO1, MCM10, and NCAPH, shown in Figure 2D; Supplementary Table S2.

### CUT&Tag sequencing analysis

**FIGURE 3:**
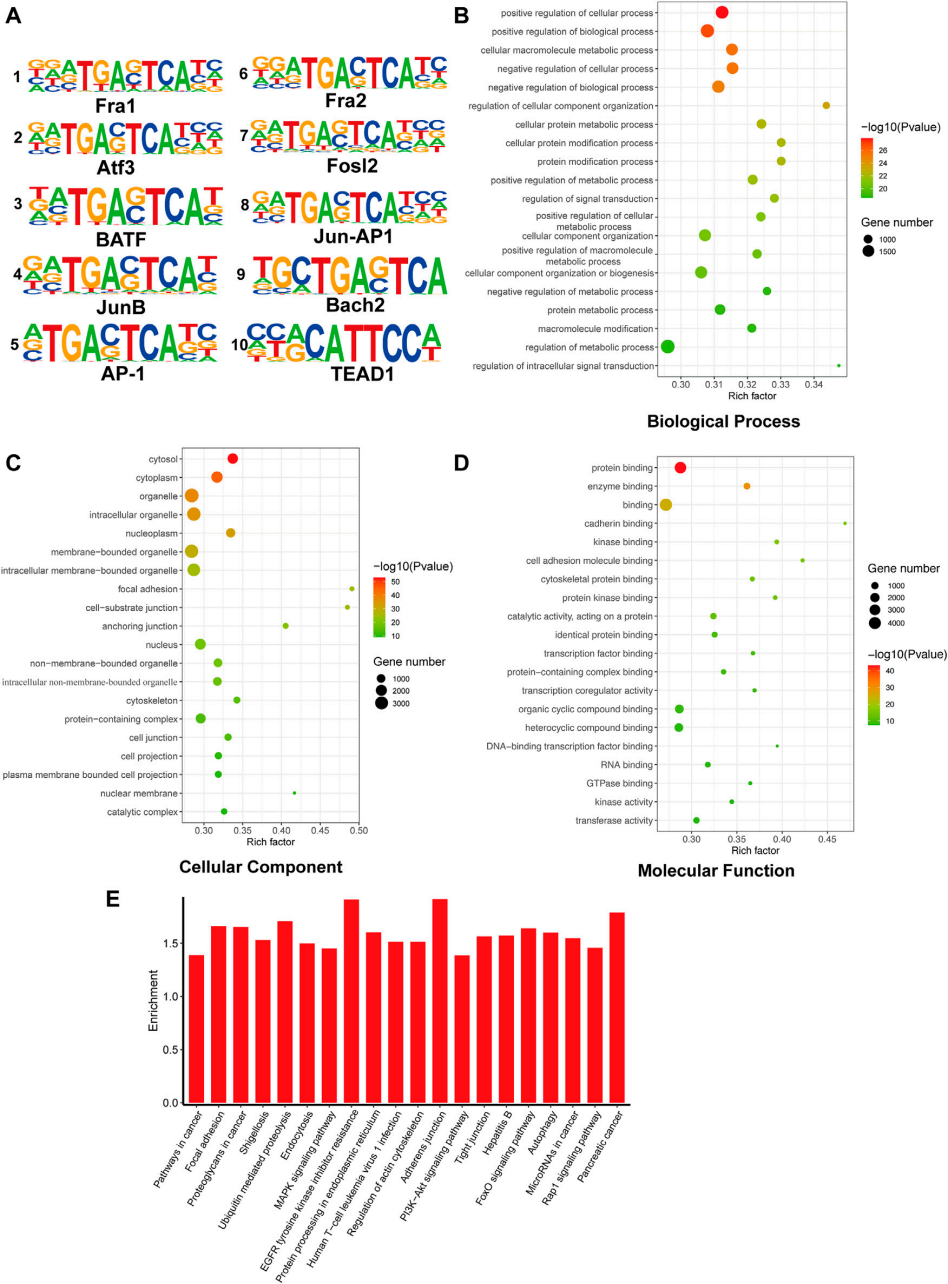
CUT&Tag seq analysis. **(A)**. The top 10 transcriptional factors binding to YAP. **(B–D)**. GO analysis of genes related to peaks. **(E)**. KEGG analysis of genes related to peaks.

As YAP is identified as a transcriptional co-activator, it plays an important role in the transcription regulatory process. To illustrate the function of YAP, immunoprecipitation for YAP CUT&Tag sequencing was performed. The Reads enrichment region is known as Peak. The heatmap of the Peak central region showed that the signals conversed near the enrichment location, indicating the data’s satisfying quality (Supplementary Figure S1A). In addition, the distribution of Peaks in functional gene regions is shown in Supplementary Figure S1B. The predicted motifs were matched with HOMOR. The top 10 known sequences of predicted motifs are listed in Figure 3A. The GO annotation analysis showed that the genes enriched in positive regulation of the cellular process, metabolism, protein binding, signal transduction process, and others (Figures 3B–D). In KEGG enrichment analysis, the genes function in focal adhesion, MAPK signaling pathway, and PI3K/Akt signaling pathway that is crucial in cell development and proliferation processes (Figure 3E).

### The cross-analysis of RNA-seq and CUT&Tag sequencing to identify the potential mechanism of the YAP target gene regulatory network

**FIGURE 4:**
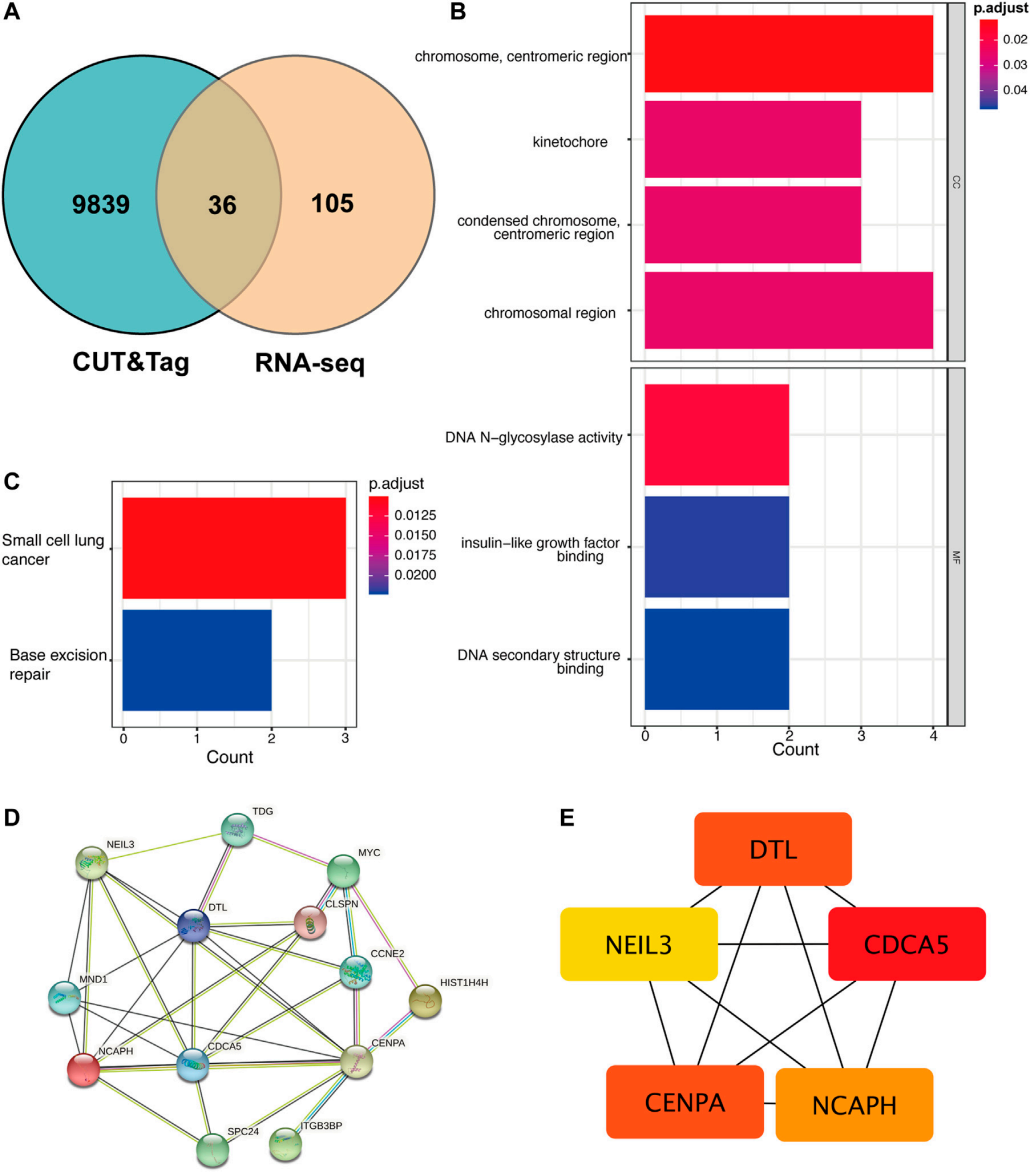
The cross-analysis of RNA-seq and CUT&Tag to identify the potential YAP target gene regulatory network mechanism. **(A)**. The Venn diagram of matching DEGs between RNA-seq and CUT&Tag seq. **(B)**. GO analysis of matching DEGs. **(C)**. KEGG analysis of matching DEGs. **(D)**. The PPI network of matching DEGs. **(E)**. Top five hub genes of matching genes. DEG: differentially expressed genes, PPI: protein-protein interaction.

Not all the DEGs in RNA-seq are YAP directly interacted with. The DNA fragments that might directly interact with YAP were analyzed to identify the regulatory mechanisms of YAP. 9,875 predicted genes are binding to YAP. After combining RNA-seq and CUT&Tag sequencing, 36 DEGs were identified to bind to YAP (Figure 4A). The interactive genes between RNA-seq and CUT&Tag are listed in Supplementary Table S3. Among these genes, 10 DEGs were up-regulated, and 26 were down-regulated. The GO annotation indicated that the DEGs enriched the DNA activity process, binding process, and chromosomal region (Figure 4B). The KEGG annotation revealed that the DEGs enriched in the base excision repair process (Figure 4C). Then, a PPI analysis was conducted to clarify the interactions between the proteins (Figure 4D). The network was further analyzed in *Cytohubba, and the* MCC method selected the top five hub genes (Figure 4E). The five hub genes were identified in Table 2.

**TABLE 2:** Top 5 in PPI network of interactive genes ranked by Maximum Climate Centrality (MCC) method.

Rank	Name	Score	Regulate
1	CDCA5	138	down
2	CENPA	134	down
2	DTL	134	down
4	NCAPH	132	down
5	NEIL3	122	down

## Discussion

YAP engages in many biological processes, such as cell proliferation, differentiation, apoptosis, and metabolism. In fibrotic diseases, YAP engages in fibroblast activation (Liu et al., 2015), wound healing process (Dey et al., 2020), and ECM remodeling (Zhang et al., 2022). Importantly, YAP can respond to mechanical cues to promote atherosclerosis, fibrosis, cardiac hypertrophy, muscular dystrophy, and cancer (Panciera et al., 2017). For example, the CRISPR/Cas9-mediated endothelial YAP knockdown attenuated the vascular plaque formation in mice, which indicates the association between YAP and atherosclerosis (Wang et al., 2016). The ECM stiffness triggered TGFβ-YAP signaling to promote kidney fibrosis (Szeto et al., 2016). In addition, ECM remodeling activated cardiac fibroblast and cardiac hypertrophy (Perestrelo et al., 2021). Furthermore, YAP also plays a pivotal role in mechanically sensing the urinary bladder. However, inflammatory responses, epithelial cell responses, smooth muscle cell hypertrophy, and ECM remodeling are all included in bladder fibrosis. Whether YAP functions in these processes are unclarified. Hence, the purpose of our study is to elaborate on the function of YAP of smooth muscle in bladder fibrosis progression.

We first infected the hBdSMCs with AAV in the current study to knock down YAP gene expression. Then RNA-seq was performed to illustrate YAP-regulated gene functions and pathways. The results revealed that YAP might engage in cell cycle regulation. With the highly development of high-throughput sequencing technologies, there comes great attention on multiple omics analyses to clarify human diseases (Yu et al., 2021). Hence, a CUT&Tag sequencing was conducted to identify the genes that directly bind to YAP. In the top 10 predicted genes, Fra-1-regulated transcription was reported to promote the proliferation process of breast cancer (Zhao et al., 2014). Studies demonstrated that the BATF-Jun family interacted with interferon regulatory factor 4 to promote lymphoid development (Li et al., 2012). In the activator protein-1 (AP-1) transcription factor family, JunB functions dually in the cell cycle. JunB was initially recognized as a cell proliferation inhibitor. However, JunB also promotes cell division (Piechaczyk and Farràs, 2008), i,e., JunB is identified as pivotal in angiogenesis (Yoshitomi et al., 2021). Like JunB, c-Jun also belongs to the AP-1 family, which involves many biological processes, such as cell proliferation, survival, apoptosis, and tissue morphogenesis (Meng and Xia, 2011). In kidney fibrosis, G2/M-arrested proximal tubular cells facilitated c-Jun signaling to promote the production of fibrotic cytokines (Yang et al., 2010). YAP often binds to the TEA-binding domain (TEAD) family of transcription enhancers to promote downstream gene expression (Kaan et al., 2017). A recent study demonstrated that TEAD1 promoted vascular smooth muscle cells (VSMCs) through solute carrier family member 5 (SLC1A5), thereby activating mTORC1 signaling to facilitate endothelium formation (Osman et al., 2019).

After interactive matching, 36 DEGs were selected. The sheltered hub genes enriched the cell cycle process, which indicated that YAP promoted hBdSMCs proliferation in response to mechanical stimuli. The PPI network stated the top five hub genes through rankings. Coincidentally, the five hub genes are all down-regulated. Cell division cycle-associated 5 (CDCA5) has been widely studied in human cancer progression. In general, CDCA5 is identified as an oncogene and has a poor prognosis for cancers (Chang et al., 2015). For instance, CDCA5-knockdown inhibited cell proliferation, migration, and clone formation in breast cancer (Hu et al., 2022). The degradation of CDCA5 also inhibits prostate cancer progression (Luo et al., 2021). CDCA5 regulates cell proliferation through various signaling. Recent research illustrated that CDCA5 activated prostate cancer and colorectal cancer cell proliferation *via* ERK signaling pathway (Shen et al., 2019; Ji et al., 2021). In addition, the ablation of CDCA5 inhibited gastric cancer cell proliferation *via* downregulating Cyclin E1 expression (Zhang et al., 2018).

Centromere Protein A (CENPA) is highly correlated with cell proliferation. The centromere is a chromatin structure that provides an assembly site for cell machinery, which is essential in cell proliferation and survival (Fukagawa and Earnshaw, 2014). Mechanistically, CENPA is assembled into the centromeric chromatin in the cell cycle to the following cell cycle and generation (Wong et al., 2020). Studies demonstrated that the expression of CENPA in cardiac progenitor cells (CPCs) decreased along with aging. The expression level of CENPA is relatively higher in the early stage of life, thereby sustaining cell proliferation, inhibiting senescence, and triggering CPCs differentiation (McGregor et al., 2014). The ablation of CENPA inhibited cell proliferation in ovarian cancer (Han et al., 2021). Interestingly, circular RNAs (circRNAs), defined as crucial cancer regulators, decreased the expression of FOXM1 and promoted the expression of CENPA and CENPB to facilitate cell cycle progression (Cheng et al., 2019).

DTL is identified as CUL4-DDB1 associated factors (DCAFs), engaged in many tumorigenesis processes. Studies revealed that DTL enhanced the proliferation and migration of cancer cells in nude mice (Cui et al., 2019). In addition, the increase of DTL indicated a poor prognosis in malignant manners of bladder cancer through the mTOR/Akt signaling cascades (Luo et al., 2022). Similarly, the non-structural maintenance of chromosomes condensing I complex subunit H (NCAPH) also facilitated cell proliferation, migration, invasion, and epithelial-to-mesenchymal transition (EMT) of cancer (Kim et al., 2020; Wang et al., 2020). And some microRNAs also target NCAPH to promote the degradation of *β*-catenin to reduce cancer stem cell maintenance (Wang et al., 2020). The fifth hub gene, the base excision repair enzyme NEIL3, plays a vital role in many biological processes, including fibrosis, lipid metabolism, tumorigenesis, and neurogenesis. For instance, in NEIL3^−/−^ heart ruptured mice, the fibroblasts and myofibroblasts increased significantly, indicating that NEIL3-dependent regulation of DNA methylation affected the fibroblast proliferation and the ECM modulation (Olsen et al., 2017). Interestingly, we have found that epigenetics alterations in cell-free DNA genome were widely distributed in multiple diseases, which might be critical in early diagnosis of fibrosis and cancer disease (Yu et al., 2020). In the smooth muscle remodeling process, the depletion of DNA glycosylase NEIL3 promoted differentiation of aortic VSMCs through the Akt signaling pathway (Quiles-Jiménez et al., 2021). NEIL3 has also been reported to mediate the lipid metabolism and macrophage function in myocardial infarction (Skarpengland et al., 2016).

Collectively, the hub genes are enriched in the processes of cell proliferation and cell survival. The question is how smooth muscle proliferation behaves in bladder fibrosis. pBOO was caused by bladder wall inflammation, hypertrophy, and fibrosis (Siregar et al., 2022). Studies demonstrated that pBOO-induced bladder fibrosis was attributed to fibrosis, smooth muscle cell proliferation and hypertrophy, and urothelium proliferation (Qiao et al., 2018). In female pBOO rats, the bladder smooth muscle progresses in hematoxylin and eosin staining and is confirmed by increased bladder mass and thickness increase (Metcalfe et al., 2010). The bladder wall thickening functions in compensatory mechanisms against pathological mechanical forces in the urinary bladder, which ultimately results in a fibrotic bladder with low capacity and high pressure. Therefore, early intervention of pBOO is necessary, and knowing how to regulate these hub genes’ functions might provide novel insights into preventing pBOO-induced fibrosis. In addition, a recent study demonstrated that computational framework for analyzing multi-omics profiles provided a novel direction for clinical diagnosis of many diseases (Yu et al., 2019). Combined analyses based on well-established tools may validate the results further.

Our study inevitably has some limitations. Although the CUT&Tag sequencing is more reliable than the common ChIP-seq, the low repeatability is still a common claw that cannot be solved to date. Hence, only two samples (Experiment and IgG) were applied for CUT&Tag sequencing. Furthermore, since bladder fibrosis is associated with several cell types, including urothelial cells, smooth muscle cells, and fibroblasts, the results from bladder smooth muscle cells can partly reflect the possible therapeutic targets. Further research on other cell types is needed.

## Conclusion

The current study identified the potential mechanisms for YAP and its interactive hub genes in urinary bladder remodeling. The role of YAP in pBOO-induced bladder fibrosis is unclear. Our research confirmed that YAP is important in bladder smooth muscle proliferation and hypertrophy. Although pBOO-induced fibrosis is complex, the therapies targeting YAP might be a potential treatment for pBOO.

## Data Availability

The datasets presented in this study can be found in online repositories. The names of the repository/repositories and accession number(s) can be found below: https://www.ncbi.nlm.nih.gov/genbank/, PRJNA856421.
