# Supplementary material for: YAP-mediated mechanotransduction in urinary bladder remodeling: Based on RNA-seq and CUT&Tag
Source: Front Genet. 2023 Jan 20;14:1106927. doi: 10.3389/fgene.2023.1106927 (PMC9895788; doi:10.3389/fgene.2023.1106927)
Supplement: Supplementary file 1 [file Table1.DOCX]

**Supplementary Materials**

**FigureS1 The quality control of CUT&Tag sequencing.
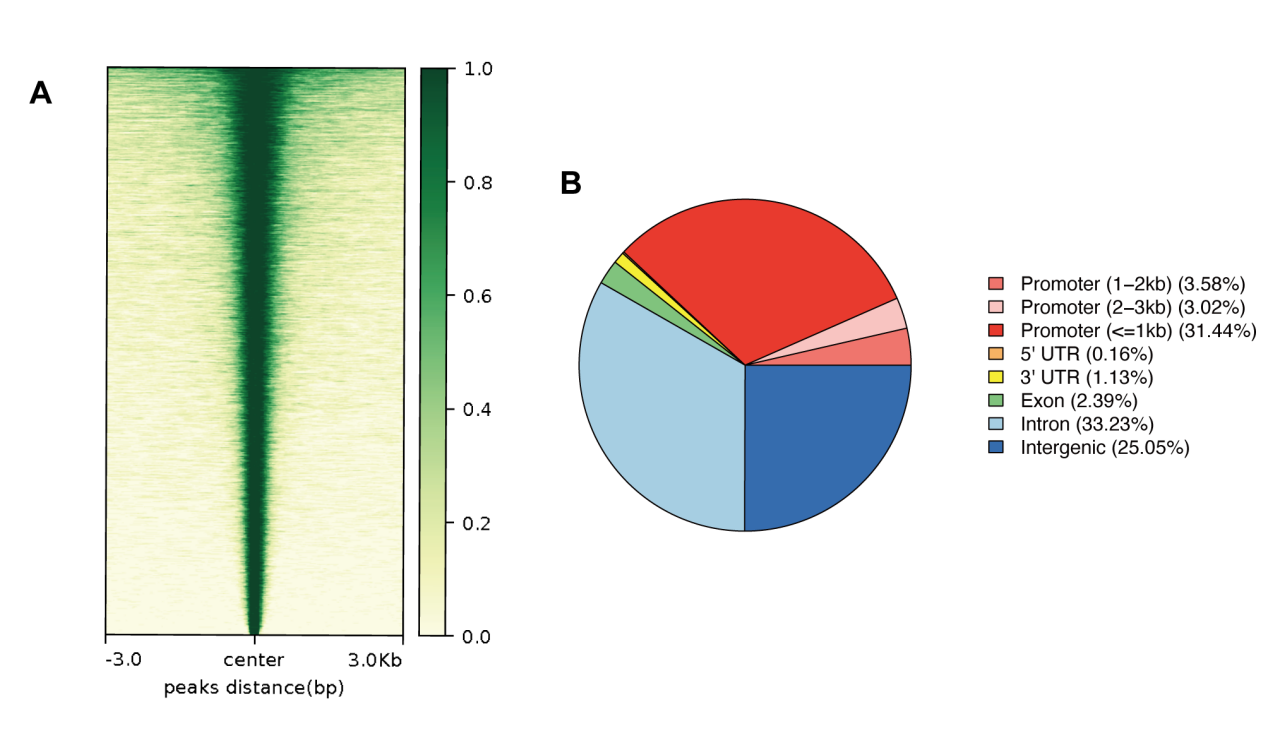
**A. The central signal heatmap of peaks. B. The distribution of peaks in functional region. Promoter: The ratio of Peak in the region from the promoter to the transcription start site, the default range of this region is 3kb upstream of the gene start to 3kb downstream of the gene start; 5'UTR: The ratio of Peak in the 5' non-coding region; 3'UTR : Peak ratio in 3' non-coding regions; Exon: Peak ratio in exons; Intron: Peak ratio in introns; Intergenic: Peak ratio in intergenic regions.

**TableS1. Quality control of RNA-seq and CUT&Tag sequencing.**

| **Type** | **Sample Name** | **Raw Reads** | **Clean Reads** | **Q20(%)*** | **Q30(%)*** | **GC Content(%)** |
| --- | --- | --- | --- | --- | --- | --- |
| CUT and TAG | EXP | 54525580 | 54081146 | 98.65 | 95.06 | 31.73 |
| CUT and TAG | IgG | 81080 | 79706 | 97.89 | 93.19 | 35.64 |
| RNA-seq | KD_1 | 59099906 | 59000070 | 98.33 | 95.15 | 49.06 |
| RNA-seq | KD_2 | 57282826 | 57169062 | 98.33 | 95.16 | 49.11 |
| RNA-seq | KD_3 | 54407232 | 54310066 | 98.45 | 95.43 | 48.94 |
| RNA-seq | NC_1 | 61413862 | 61294520 | 98.32 | 95.14 | 48.92 |
| RNA-seq | NC_2 | 53732138 | 53645040 | 98.39 | 95.24 | 48.80 |
| RNA-seq | NC_3 | 59844848 | 59751514 | 98.33 | 95.11 | 48.61 |

*Q20 represents the ratio between the Reads with the accuracy of bases above 99% and total Reads; Q30 represents the ratio between the Reads with the accuracy of bases above 99.9% and total Reads. KD, Knock down; NC, nature control.

**TableS2. Top 5 in PPI network of RNA-seq ranked by MCC method.**

| **Rank** | **Name** | **Score** |
| --- | --- | --- |
| 1 | RAD51AP1 | 2036640 |
| 2 | CDCA5 | 2036526 |
| 3 | EXO1 | 2036298 |
| 4 | MCM10 | 2021052 |
| 5 | NCAPH | 1996200 |

MCC,Maximum Climate Centrality

**TableS3. The interactive genes between RNA-seq and CUT&Tag sequencing.**

| **Gene ID** | **Gene Name** | **Log2FC** | **Padj*** | **Regulate** |
| --- | --- | --- | --- | --- |
| ENSG00000092853 | CLSPN | -1.249739965 | 1.18E-37 | down |
| ENSG00000109674 | NEIL3 | -1.084692358 | 7.76E-07 | down |
| ENSG00000115163 | CENPA | -1.222711126 | 0.023739069 | down |
| ENSG00000121152 | NCAPH | -1.02305293 | 4.14E-17 | down |
| ENSG00000121211 | MND1 | -1.125082527 | 1.50E-10 | down |
| ENSG00000122376 | SHLD2 | -1.216862024 | 1.18E-92 | down |
| ENSG00000136144 | RCBTB1 | -1.04402106 | 9.71E-51 | down |
| ENSG00000138448 | ITGAV | -1.120115672 | 1.70E-141 | down |
| ENSG00000139112 | GABARAPL1 | -1.284554326 | 1.18E-11 | down |
| ENSG00000139372 | TDG | -1.174942944 | 8.73E-58 | down |
| ENSG00000141933 | TPGS1 | -1.923841877 | 0.004374417 | down |
| ENSG00000142856 | ITGB3BP | -1.116485321 | 1.45E-29 | down |
| ENSG00000143476 | DTL | -1.070825499 | 1.29E-12 | down |
| ENSG00000146670 | CDCA5 | -1.283019961 | 9.66E-17 | down |
| ENSG00000148841 | ITPRIP | -2.408022075 | 3.95E-64 | down |
| ENSG00000158246 | TENT5B | -1.062130039 | 9.34E-08 | down |
| ENSG00000161888 | SPC24 | -1.134181364 | 9.16E-09 | down |
| ENSG00000165244 | ZNF367 | -1.924989886 | 3.13E-24 | down |
| ENSG00000175305 | CCNE2 | -1.26492422 | 1.89E-16 | down |
| ENSG00000178460 | MCMDC2 | -1.064332195 | 0.031259936 | down |
| ENSG00000187187 | ZNF546 | -1.871693432 | 4.82E-05 | down |
| ENSG00000189362 | NEMP2 | -1.10543897 | 6.03E-11 | down |
| ENSG00000213853 | EMP2 | -1.103154786 | 2.95E-16 | down |
| ENSG00000216866 | RPS2P55 | -1.197160019 | 0.041421554 | down |
| ENSG00000229124 | VIM-AS1 | -1.046613835 | 0.005972472 | down |
| ENSG00000260643 | AC092718.3 | -1.56809585 | 0.01570349 | down |
| ENSG00000007314 | SCN4A | 1.107893248 | 0.000986222 | up |
| ENSG00000136997 | MYC | 3.53540032 | 0.003839029 | up |
| ENSG00000141404 | GNAL | 2.117277467 | 3.83E-11 | up |
| ENSG00000152127 | MGAT5 | 1.069399831 | 0.003634386 | up |
| ENSG00000158406 | HIST1H4H | 1.327502895 | 0.00106951 | up |
| ENSG00000163618 | CADPS | 1.046222243 | 0.00017993 | up |
| ENSG00000170801 | HTRA3 | 1.234355134 | 8.50E-57 | up |
| ENSG00000177453 | NIM1K | 1.407592538 | 3.00E-15 | up |
| ENSG00000204778 | CBWD4P | 1.725271203 | 7.24E-13 | up |
| ENSG00000260822 | AC004656.1 | 1.212202854 | 1.77E-10 | up |

*Padj, adjusted P value.
